# Acupuncture: A Promising Non‐Pharmacological Approach to Parkinson's Disease Management

**DOI:** 10.1002/brb3.71438

**Published:** 2026-04-22

**Authors:** Xi‐Chen Wu, Yi‐Yue Dong, Yu‐Chen Ying, Ping Yin, Yue‐Lai Chen

**Affiliations:** ^1^ LongHua Hospital Shanghai University of Traditional Chinese Medicine Shanghai China

**Keywords:** acupoint, acupuncture, molecular mechanism, Parkinson's disease

## Abstract

**Purpose:**

This review aims to elucidate the molecular mechanisms underlying the neuroprotective effects of acupuncture in preclinical models of Parkinson's disease (PD).

**Finding:**

In PD animal models, acupuncture inhibits oxidative stress by upregulating nuclear factor erythroid 2‐related factor 2 (Nrf2)/antioxidant response element (ARE), superoxide dismutase (SOD), and glutathione peroxidase (GSH‐Px) while reducing malondialdehyde (MDA) and lipid peroxidation. It regulates autophagy either independently of mammalian target of rapamycin (mTOR) or via mTOR activation, promoting alpha‐synuclein (α‐synuclein) clearance. Acupuncture also suppresses apoptosis (modulating Bcl‐2‐associated X protein (Bax)/B‐cell lymphoma 2 (Bcl‐2)) and pyroptosis (inhibiting NLR family pyrin domain containing 3 (NLRP3) inflammasome and gasdermin D (GSDMD)). It enhances neurogenesis through brain‐derived neurotrophic factor (BDNF)/extracellular signal‐regulated kinase (ERK)/cyclic adenosine monophosphate (cAMP) response element‐binding protein (CREB) and glial cell line‐derived neurotrophic factor (GDNF) signaling, promoting neural stem cell proliferation and differentiation. Furthermore, acupuncture reduces neuroinflammation by decreasing microglial activation, cyclooxygenase‐2 (COX‐2), tumor necrosis factor‐alpha (TNF‐α), and interleukin‐1 beta (IL‐1β). It also modulates gut microbiota composition (e.g., increasing butyrate‐producing bacteria like Butyricimonas and reducing pro‐inflammatory Erysipelotrichaceae and Bacteroides) and influences lipid metabolism, thereby mitigating dopaminergic neuron loss and motor deficits.

**Conclusion:**

Preclinical evidence demonstrates that acupuncture exerts multi‐target neuroprotective effects against PD through pathways involving oxidative stress, autophagy, apoptosis/pyroptosis, neurogenesis, neuroinflammation, and gut microbiota‐lipid metabolism crosstalk. However, limitations include a focus on preventive rather than reversal effects, lack of long‐term efficacy data, and heterogeneity in acupoint selection. Further mechanistic and standardization studies are warranted.

## Graphical abstract

The mechanism of acupuncture treatment of PD models involves oxidative stress, intestinal flora, neuroinflammation, autophagy, neurogenesis, pyroptosis, and apoptosis.

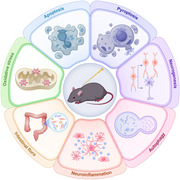

## 1 Introduction

Parkinson's disease (PD) is a chronic progressive neurodegenerative disorder that frequently affects middle‐aged and older persons, and its prevalence increases with age (Pringsheim et al. 2014). Postural instability, bradykinesia, resting tremor, and myotonia are among the main clinical manifestations (Pringsheim et al. 2014). Besides motor issues, PD progression can lead to non‐motor symptoms like cognitive impairment, mood disorders (apathy, depression, anxiety, and hallucinations), and autonbomic dysfunctions (constipation, hypothermia, and sleep disorders) (F.‐P. Chen et al. 2015). The prevalence of PD is 0.3%, making it the second most frequent neurological disease after Alzheimer's disease (AD) (Cheng 2017). Unfortunately, even with treatment, PD patients' physical state deteriorates, especially in the majority of PD patients with a disease duration of more than 20 years (Cheng 2017; Kalia and Lang 2015; Robbins and Cools 2014). More than four‐fifths of people with PD eventually develop dementia (Aarsland et al. 2010). The cost of treatment places a huge financial demand on patients and their families, exceeding $14 billion per year (Kowal et al. 2013).

Pathologically, PD is characterized by nerve degeneration in the substantia nigra (SN), the deposition of α‐synuclein (α‐syn), and the development of Lewy bodies. (Simon et al. 2020). Various treatment modalities are available for PD, including pharmacologic, surgical, and exercise therapies. Pharmacologic treatment is typically the initial choice, with dopaminergic and anticholinergic drugs being the most commonly prescribed medications (Kalia and Lang 2015). These drugs have demonstrated rapid and effective control of patients' initial symptoms (Kalia and Lang 2015). However, long‐term therapy with levodopa is frequently associated with motor fluctuations, which can arise from factors such as discontinuous administration, short half‐life, poor bioavailability, and a narrow therapeutic window (Tambasco et al. 2018). Non‐pharmacological interventions have significantly transformed the management of PD. A range of supplements, including medicinal plants (Rajasankar et al. 2009; Mahboubi et al. 2016), vitamins (Rascol et al. 2015; Suzuki et al. 2013), omega‐3 fatty acids (Bousquet et al. 2011; Bousquet et al. 2011), and probiotics (Borzabadi et al. 2018; Tamtaji, Taghizadeh, et al. 2019), have demonstrated efficacy in controlling PD and age‐related conditions. For instance, probiotic supplementation has been found to mitigate oxidative stress and inflammatory factors in individuals with PD, leading to improvements in certain clinical manifestations (Jin et al. 2024). Various forms of exercise have been shown to be efficacious in managing the clinical manifestations of PD within the realm of non‐pharmacological interventions (De Melo et al. 2018; Kwok et al. 2017; Luna et al. 2018). For example, high‐intensity exercise has been shown to effectively manage motor symptoms during the initial phases of PD (Fisher et al. 2008). Sharma et al. found that yoga has the potential to enhance the quality of life for individuals with PD (Sharma et al. 2015). Various surgical interventions, such as deep brain stimulation (DBS), ablation or lesion surgery (pallidotomy, thalamotomy), and dopaminergic drug infusion devices, are accessible for managing motor complications associated with PD (Simon et al. 2020). The management of PD can be classified into early and advanced stages. Early PD represents the initial phase of the illness, during which symptoms may have minimal impact on daily functioning. An analysis of 15 years of treatment outcomes in Romanian patients with PD indicates that there is a growing body of evidence supporting the use of levodopa‐sparing medications in the management of the disease (Szász et al. 2019). Alternative therapies can be considered alongside other antiparkinsonian medications in appropriate clinical scenarios to reduce levodopa doses (Szász et al. 2019). Advanced PD refers to the later stages of the condition, which are characterized by severe motor complications and a range of non‐motor symptoms. Treatment options for advanced PD include levodopa‐carbidopa enteric gel, continuous subcutaneous apomorphine infusion, DBS, radiofrequency ablation, stereotactic radiosurgery, and magnetic resonance imaging‐guided focused ultrasound (Serva et al. 2022). Among these invasive treatments, DBS has the most clinical evidence; however, it is complex, costly, and carries a risk of serious complications. Despite the availability of these treatments, no single intervention has been proven to halt the progression of PD (Bloem et al. 2021). Hence, the selection of alternative therapies aimed at slowing the progression of PD is crucial.

Acupuncture, a traditional Chinese medicine (TCM) therapy with a long history, originated in China. While TCM theory suggests that acupuncture works by stimulating specific areas (acupoints) on the meridians (that is, the pathways through which “qi” flows) to regulate physiological functions, modern science provides increasing evidence for the biological effects of acupuncture. This evidence indicates that acupuncture stimulates reflexes, activates peripheral nerves transmitting sensory information from the spinal cord to the brain, and activates peripheral autonomic neural pathways to modulate physiological functions (S. Liu et al. 2021; Ma 2020; Sato 1997). Recent researches have shown the effectiveness of acupuncture in treating various central nervous system (CNS) disorders, including PD (Kwon et al. 2021), stroke (T.‐Y. Choi et al. 2022), pain (Lam et al. 2022), spinal cord injury (J. Tan et al. 2022), and major depression (M. Kim et al. 2022). Studies have highlighted the benefits of electroacupuncture (EA) as a non‐pharmacological treatment for PD, including improvements in clinical symptoms, reductions in drug side effects, delays in disease progression, and enhancements in quality of life (Tamtaji, Naderi Taheri, et al. 2019; Shulman et al. 2002; L. C. S. Tan et al. 2006; X. Wang, Liang, et al. 2008). Furthermore, these studies indicate that both acupuncture (Noh et al. 2017) and EA (K. Li et al. 2023) positively impact motor symptoms in PD patients, potentially decreasing the need for anti‐PD medications and mitigating their associated side effects (J. Huang et al. 2020). The therapeutic mechanisms of acupuncture are associated with reduced oxidative stress, protein aggregation, impaired autophagy, neuroinflammation, and so on (Ko et al. 2019). In this review, we investigate the possible mechanisms of acupuncture for treating PD, with the hope of providing new insights for its clinical application in reversing dopaminergic neuronal degeneration and slowing the progression of PD.

## 2 Molecular Mechanisms Underlying the Neuroprotective Effects of Acupuncture on PD

### 2.1 Inhibition of Oxidative Stress

Acupuncture in 6‐hydroxydopamine (6‐OHDA) rats has been shown to elevate levels of SOD and glutathione peroxidase (GSH‐Px), while simultaneously reducing MDA levels and mitigating oxidative stress (Y.‐P. Yu et al. 2010). Additionally, acupuncture at the GB34 acupoint in the striatum of 1‐methyl‐4‐phenyl‐1,2,3,6‐tetrahydropyridine (MPTP) mice resulted in increased activities of SOD and CAT (H. Wang et al. 2011). Electrical stimulation of the ST36 and SP6 acupoints caused a decrease in H_2_O_2_ and MDA levels, while enhancing SOD, GSH, and GSH‐Px levels. (Wattanathorn and Sutalangka 2014).

The endogenous antioxidant defense system is regulated by nuclear factor erythroid 2‐related factor 2 (Nrf2), a crucial transcription factor in maintaining redox homeostasis. By binding to the antioxidant response element (ARE), Nrf2 can activate downstream antioxidant genes (Sivandzade et al. 2019). Nrf2‐deficient mice exhibit hypersensitivity to PD‐generating neurotoxins (P.‐C. Chen et al. 2009). One study found that EA reversed inhibition of the Nrf2/ARE system in a PD mouse model, suggesting that EA significantly exerts antioxidant effects by increasing Nrf2 and ARE expression (Lv et al. 2015). Similarly, another study reported a similar upregulation of Nrf2/ARE expression by EA in A53T mice (Deng et al. 2015).

Research shows that lipid peroxidation, caused by elevated free radicals, is more pronounced in PD patients (Agil et al. 2006; De Farias et al. 2016). Mitochondria are especially vulnerable to oxidative damage, resulting in structural alterations, including mitochondrial swelling or shrinkage, loss of mitochondrial cristae, and mitochondrial vacuolization (Zuo et al. 2022). Acupuncture at GB34 in PD mice demonstrated the capability to mitigate the MPTP‐induced escalation of mitochondrial abnormalities, reduce the density of prominent vesicles, enhance the thickness of the myelin sheath (thereby affecting impulse conduction), and restore both the horseshoe structure of the Golgi apparatus and the integrity of the mitochondrial envelope and cristae (Zuo et al. 2022). These alterations collectively contributed to the restoration of organelle structure and the enhancement of synaptic and axonal functions. Fourier Transform Infrared Spectroscopy microspectroscopy revealed that acupuncture reduced the lipid ratio in dopaminergic neurons in the brains of PD mice, inhibited lipid peroxidation, and thus reduced the damage to dopaminergic neurons caused by oxidative stress (Zuo et al. 2022).

### 2.2 Regulation of Autophagy

Numerous lines of evidence indicate that various signaling pathways play a role in the regulation of autophagy (Q. Ma et al. 2023; Braicu et al. 2022; Pan and Valapala 2022). The mammalian target of rapamycin (mTOR) is a key player in autophagy regulation (T. Huang et al. 2024). Autophagy aids in the removal of toxic protein aggregates and dysfunctional mitochondria in neurons, which is pertinent to the advancement of neurodegenerative disorders like AD and PD (Fujikake et al. 2018; Sarkar 2013). Multiple studies have demonstrated that the activation of autophagy results in enhanced clearance of neuronal α‐syn, ultimately leading to inhibition of neurodegeneration in PD (Webb et al. 2003; Cookson 2009; Abulimiti et al. 2022). Rapamycin has been identified as a stimulator of autophagy, yet it has been shown to have negative impacts on glucose metabolism, exacerbate diabetes, and decrease the permeability of the blood‐brain barrier (BBB) (Chi et al. 2017). Consequently, there is a pressing need to explore alternative therapeutic approaches. While DBS in the subthalamic nucleus has been found to activate autophagy (Du et al. 2018), its intricate procedure, high cost, and potential for severe complications hinder its widespread adoption. Conversely, acupuncture is a widely utilized complementary therapy with minimal adverse effects. According to recent research, acupuncture at the GB34 point has been shown to enhance the autophagic clearance of α‐syn in MPTP mice via a pathway that operates independently of mTOR (Tian et al. 2016). Additionally, Wei‐Ti Hsu et al. showed that EA stimulates neuronal autophagy initiation, autophagosome formation, and autophagic flux/substrate degradation in nigral, striatal, hippocampal, and cortical neurons of MPTP mice (Hsu et al. 2020). Specifically, it was observed that EA increased the expression levels of protective genes Beclin 1, phosphatase and tensin homolog‐induced putative kinase 1 (PINK1), and protein deglycase DJ‐1 (DJ‐1) in PD mice within the striatum, hippocampus, and cortex (Hsu et al. 2020). However, another study in a rat model of PD with depression suggests that acupuncture may alleviate symptoms by activating the mTOR signaling pathway, inhibiting excessive autophagy in the striatum, and modulating synaptic plasticity, thereby increasing the content of monoamine neurotransmitters (Ning et al. 2023). This suggests that the use of EA bidirectionally modulates neuronal autophagy and holds therapeutic potential for treating neurodegenerative diseases in the striatum, hippocampus, or cortex.

### 2.3 Inhibition of Apoptosis and Pyroptosis

The prevalent forms of programmed cell death encompass autophagy, apoptosis, pyroptosis, necroptosis, ferroptosis, and cuproptosis (Moujalled et al. 2021). In the current study, excluding autophagy mentioned above, apoptosis and pyroptosis were incorporated into the molecular mechanisms underlying acupuncture's effects in the treatment of PD.

Neuronal damage and apoptosis have been reported in several studies on PD (Tatton et al. 2003; Tatton 2000; Lev et al. 2003). Jang et al. discovered that manual acupuncture (MA) at GB34 in MPTP mice suppressed the increase in Bcl‐2‐associated X protein (Bax), nuclear factor kappa‐light‐chain‐enhancer of activated B cells (NF‐κB) and tumor necrosis factor‐alpha (TNF‐α) expression while restoring the expression of anti‐apoptotic regulators such as Bcl‐2 (Jang et al. 2020). Similar findings were reported in another study on EA treatment at GB34 and LR3 (Lin et al. 2017). ST36 and SP6 are also commonly used acupuncture points for treating PD patients (Eng et al. 2006). Researchers observed that 100 Hz EA stimulation of MPTP‐lesioned mice at ST36 and SP6 elevated the Bcl‐2/Bax ratio, resulting in anti‐apoptotic effects (H. Wang et al. 2013). Additionally, animal studies on EA targeting the tremor control region of the head in chorea have demonstrated that EA enhances motor coordination and upper limb grip strength in MPTP mice, while inhibiting midbrain Bax expression and promoting Bcl‐2 expression (Geng et al. 2024).

Inflammasome is initially characterized as an integral components of the immune response, activated by immune cells in reaction to various harmful stimuli (Broz and Dixit 2016). Dysregulation of its activation has been associated with the onset and progression of various age‐related pro‐inflammatory diseases, including diabetes, atherosclerosis, gout, and neurodegenerative disorders such as AD and PD (H. Guo et al. 2015). Accumulating evidence indicates that NLR family pyrin domain containing 3 (NLRP3) inflammasomes are activated by gasdermin D (GSDMD), leading to pyroptosis in dopaminergic neurons within PD mouse models and MPTP or MPP^+^ cell models (X. Ma, Chen, et al. 2021; Rui et al. 2020). GSDMD is a member of the Gasdermin family, which also includes Gasdermin A, Gasdermin B, Gasdermin C, and DFNA5/Gasdermin E (GSDME). Notably, GSDME is hypothesized to function as a dual switch between apoptosis and pyroptosis, a process regulated by dynamic variations in Caspase‐3 activity (Rogers et al. 2019; B. Wang et al. 2023). GSDME induces cell pyroptosis by integrating its cleaved N‐terminal fragment (GSDME‐N) into the cell membrane (H. Lu et al. 2018). It also facilitates apoptosis by permeabilizing the mitochondrial membrane, thereby promoting the release of cytochrome c. During cellular damage, GSDME has been reported to activate high mobility group box 1 (HMGB1), which functions as a damage‐associated molecular pattern (DAMP) and accelerates inflammatory processes (Liao et al. 2022). Furthermore, recent studies on patients with anti‐NMDA receptor encephalitis suggest that serum Gasdermin proteins may serve as biomarkers for CNS diseases and are implicated in the pathological processes of these conditions (Rogers et al. 2019; H. Lu et al. 2018; Liao et al. 2022). There is evidence that GSDMD can cause inflammation in PD and that inhibiting the inflammasome can prevent α‐syn pathology and degeneration of dopamine neurons in PD mice (B. Wang et al. 2023; Gordon et al. 2018). L. Guo et al. (2024). demonstrated that EA treatment at GV16, LR3, and ST36 effectively inhibited the activation of the colonic NLRP3 inflammasome, reduced the mRNA expression levels of Bax and Caspase‐3, and enhanced the expression of glial cell line‐derived neurotrophic factor (GDNF). These findings suggest that EA may facilitate the repair of the intestinal barrier in PD mouse models by mitigating pyroptosis through the suppression of NLRP3 activation and the promotion of GDNF expression. Furthermore, EA treatment ameliorated MPTP‐induced reductions in white blood cell, red blood cell, hemoglobin, mean corpuscular hemoglobin concentrations, and lymphocyte counts, which are quantitatively diminished in the blood of PD patients (L. Guo et al. 2024; Maruyama et al. 1997). Notably, decreased lymphocyte counts are correlated with an elevated risk of PD (J. Lu et al. 2015). These findings suggest that EA intervention may mitigate hematological impairments in PD mice by modulating the neuroimmunoinflammatory network. Current research indicates a close association between necrotic apoptosis and ferroptosis with PD. However, this relationship has not been adequately explored in the context of acupuncture studies, necessitating further investigation in this area (Z.‐L. Wang et al. 2022; Oñate et al. 2020).

### 2.4 Promotion of Neurogenesis

Studies have reported a significant reduction in subventricular zone (SVZ) neural progenitor cells (NPCs) in patients with PD (Höglinger et al. 2004). Moreover, the non‐motor symptoms of PD, including anxiety, depression, and hypothermia, may be partially dependent on proper olfactory processing and hippocampal function, suggesting a relationship with adult neurogenesis (Marxreiter et al. 2013). Levodopa, the most prescribed medication drug for PD, has been shown to increase the number of proliferating neural stem cells (NSCs) in the SVZ, indicating a potential role in neurogenesis (O'Sullivan et al. 2011). Enhanced neurogenesis is proposed to be effective in the treatment of PD. Therefore, the restoration of neurogenesis may potentially alleviate symptoms in patients with PD.

As acupuncture stimulates the proliferation and differentiation of NSCs in the brain, it activates and promotes adult neurogenesis (Y. R. Kim et al. 2014; Ahn et al. 2016). Specifically, stimulating certain acupoints via acupuncture or EA has been shown to enhance brain cell proliferation and neuronal proliferation in specific regions: ST36 (E.‐H. Kim et al. 2002), GV20 (HWANG et al. 2010), PC6 (B. Lee et al. 2009), HT7 (Park et al. 2002), CV17, CV12, CV6, SP10 (H. Cheng et al. 2008), LI11, TE5, GB30 (Gao et al. 2011), GV16, GV8 (Z.‐J. Yang et al. 2005), CV4, CV6, CV24 (Z. Yang et al. 2008), and the stomach point of auricular acupoints (E.‐H. Kim et al. 2001). Recent studies have shown that EA and acupuncture at ST36 and GV20 stimulate adult neurogenesis, with EA being more effective than acupuncture (HWANG et al. 2010; HWANG et al. 2010). Research into the underlying mechanisms has indicated that EA significantly enhances neuroblast plasticity via phosphorylated cyclic adenosine monophosphate (cAMP) response element‐binding protein (pCREB) and brain‐derived neurotrophic factor (BDNF) activation in the DG (HWANG et al. 2010). Gao et al. found that the effects of EA on neurogenesis at ST36, LI11, TE5, and GB30 persisted for up to 4 weeks after the last treatment (Gao et al. 2011). This suggests that EA has a prolonged therapeutic effect on promoting neurogenesis in adults. Additionally, EA at GV20 has been shown to induce adult neurogenesis in both the hippocampal dentate gyrus (DG) and striatum, with the effects of short‐term EA treatment lasting for at least 26 days (S. Cheng et al. 2009). The transcription factor cAMP response element‐binding protein (CREB), which regulates BDNF transcription, can be up‐regulated by EA at ST36 and GV20 (Zhu et al. 2004; Z.‐L. Wang, Cheng, et al. 2008).

In medial forebrain bundle axotomy (MFB‐axo) PD model, stimulation of the commonly used acupoints GV20 and GV14 has been reported to activate BDNF and GDNF (Liang et al. 2003; Liang et al. 2002; X.‐Y. Liu et al. 2004). This stimulation also prevented the degeneration of dopaminergic neurons and attenuated depression (Liang et al. 2003; Liang et al. 2002; X.‐Y. Liu et al. 2004). Additionally, 2 Hz EA at GB34 enhanced neurogenesis in the SVZ of PD mice, as well as the expression of BDNF and extracellular signal‐regulated kinase (ERK) in the striatum, suggesting that EA may promote neurogenesis in the SVZ through activation of the BDNF/ERK signaling pathway (Y. Lee et al. 2023). The results showed that EA treatment enhanced the survival of nigrostriatal dopaminergic neurons and prevented the loss of striatal dopaminergic content by upregulating the expression of BDNF, GDNF, and related signaling factors such as CREB, Akt, and Pitx3, thereby improving locomotor symptoms in PD model mice (Y. Lee et al. 2023). Substantial changes in the levels of NTFs, particularly in the nigrostriatal pathway, are widely recognized as robust survival factors for dopaminergic neurons in PD patients (Nam et al. 2013). Previous studies have shown that BDNF levels are significantly reduced in the serum and cerebrospinal fluid of PD patients (Scalzo et al. 2010). Additionally, BDNF receptor signaling plays a crucial role in the survival and development of nigrostriatal dopaminergic neurons and is involved in nigrostriatal neuronal dysfunction in PD (Nie et al. 2015; Murer et al. 2001; Benraiss et al. 2012). The beneficial effects of GDNF on nigrostriatal and striatal neurons in PD have also drawn considerable attention (Patel et al. 2013). Another study has indicated that intracranial delivery of GDNF may be helpful in the treatment of PD (Kumar et al. 2015). In spite of BDNF's higher levels in the brain, it is more prevalent in the hippocampus and cerebral cortex, whereas GDNF is primarily expressed in dopaminergic structures like the striatum (Ibáñez and Andressoo 2017). In this research, a significant increase in BDNF, GDNF, and their associated receptors (TrkB and GFR‐1) was observed following EA treatment in the SN and striatum. As previously reported, striatal BDNF and GDNF levels increased more than those in the SN. Further, striatal dopaminergic nerve endings had more BDNF‐ and GDNF‐positive cells and their receptors than SN nerve endings (Ibáñez and Andressoo 2017; Oo et al. 2005).

NTFs such as BDNF and GDNF, whose transcription is influenced by CREB, are associated with behavioral and histological recovery in PD models (L. Wang et al. 2017; Cen et al. 2006; Pruunsild et al. 2011). PI3K/Akt and MAPK, which are critical intracellular mediators of BDNF and GDNF, are activated by BDNF and GDNF (Baydyuk et al. 2011; Francardo et al. 2014). Pitx3 regulates GDNF and interacts with BDNF, acting as a key transcription factor essential for dopaminergic neuronal survival and protection from neurotoxic damage. Thus, GDNF functions upstream of Pitx3, and Pitx3‐mediated BDNF synthesis is crucial for dopaminergic neuron survival and protection (L. Li et al. 2009). The stimulation of sensory neurons by EA enhances glutamate release, thus increasing glutamate receptor activation (Ryu et al. 2008). Specifically, glutamate receptors such as NMDA receptors initiate intracellular kinase cascades via calcium (Ca^2+^) influx, which in turn activates transcription factors including CREB, Activator Protein‐1 (AP‐1), and NF‐κB (Sugiyama et al. 2007; Vuong et al. 2015; Tsunoda et al. 2016). At the transcriptional level, these factors promote the expression of BDNF and GDNF (Pruunsild et al. 2011; Hisaoka et al. 2008). Furthermore, the activity of BDNF and GDNF receptors inhibits neuronal apoptosis through the phosphatidylinositol 3‐kinase (PI3K)/protein kinase B (Akt) signaling pathway and modulates interactions with the transcription factor Pitx3 (Baydyuk et al. 2011; Peng et al. 2011). Therefore, EA treatment has the potential to stimulate the production of NTFs, including BDNF and GDNF, in both neuronal and glial cells through the excitatory neurotransmitter glutamate, analogous to activity‐dependent therapeutic interventions (Pałasz et al. 2017). Furthermore, should acupuncture predominantly enhance the expression of BDNF and GDNF, these neurotrophic factors (NTFs) could subsequently activate shared intracellular phosphorylated signaling pathways via their respective receptors (Airaksinen and Saarma 2002). This implies that the expression of BDNF and GDNF induced by acupuncture may not occur through independent NTFs receptor signaling. Instead, it may involve the expression of various NTFs and the activation of receptors that share common intracellular signaling mechanisms, ultimately converging on common transcription factors such as CREB (Zhao et al. 2008). Thus, acupuncture stimulates or promotes the production of NTFs, which may function as autocrine or paracrine signals to enhance the proliferation and differentiation of NSCs and NPCs into mature neuronal cells, thereby improving cell survival. This process potentially facilitates the functional integration of newly generated neurons into the CNS, contributing to functional recovery. Consequently, acupuncture that promotes neurogenesis may represent a promising therapeutic strategy for PD.

### 2.5 Suppression of Neuroinflammation

As a response to potential threats, inflammation recruits diverse immune cells to the injured area. Neuroinflammation, distinct from inflammation in peripheral tissues, pertains to inflammatory processes within the CNS (Mukhara et al. 2020). This phenomenon is implicated in a number of neurological and psychiatric disorders, such as AD, PD, and depression (Biswas 2023; Frank‐Cannon et al. 2009; Troubat et al. 2021).

The brain's resident immune cells, microglia and astrocytes, are integral in response to various stimuli. Microglia can be activated through physiological changes known as “response states” and are involved in the release of inflammatory factors such as TNF‐α, Interleukin (IL)‐23, IL‐1β, IL‐6, IL‐18, and interferon‐γ (Q. Liu et al. 2022). Chronic neuroinflammatory responses are frequently linked to neurodegenerative diseases, with conditions like α‐syn misfolding, mitochondrial dysfunction, and immune‐related gene polymorphisms being identified as potential causes of chronic neuroinflammation (Hensley et al. 2006; Faustini et al. 2019). Recent research has focused on neuroinflammation as a potential pathology of PD. Age‐related disruptions in the brain's internal homeostasis can lead to decreased activation of survival mechanisms, resulting in heightened pro‐inflammatory cytokine production in cases of central or peripheral nervous system (PNS) dysfunction (Xanthos and Sandkühler 2014). The significance of these cytokines should not be overlooked, as they are recognized as key contributors to neurodegeneration. Research has identified increased levels of pro‐inflammatory factors (IL‐2, IL‐10, IL‐4, IL‐6, and TNF‐α) in the serum of patients with PD (Brodacki et al. 2008). Nevertheless, the causal relationship between neuroinflammation and PD remains uncertain.

In the MPTP mice model, the application of EA to the ST36 and SP6 at a frequency of 100 Hz for 30 min per day resulted in a reduction in the activation of microglia and astrocyte proliferation in the striatum and midbrain, leading to an improvement in motor function in the mice (Lv et al. 2015). Additionally, a separate study demonstrated that EA at the KI3 acupoint modulated neuroinflammation markers (IL‐1β, IL‐6, and TNF‐α) in PD dementia mice via the transient receptor potential V1, resulting in enhanced cognitive flexibility in the mice (Tsai et al. 2021). In rats subjected to MFB‐axo, 24 days of acupuncture stimulation at Du14 and Du21 significantly attenuates microglial activation, reduces the upregulation of TNF‐α and IL‐1β mRNA expression in the substantia nigra pars compacta (SNpc), and mitigates the loss of dopaminergic neurons induced by MFB‐axo resection (X.‐Y. Liu et al. 2004). Recent research has indicated that the pathogenesis of PD begins with the generation and movement of α‐syn in the gastrointestinal tract, potentially leading to brain neuroinflammation associated with damage to the gastrointestinal barrier (Klingelhoefer and Reichmann 2015). Furthermore, α‐syn accumulation in the enteric nervous system has the ability to travel to the brain via the vagus nerve, triggering microglial activation and exacerbating neuroinflammation (De Virgilio et al. 2016). X. Ma, Wang, et al. (2021) demonstrated that EA can prevent dysfunction of the intestinal barrier by regulating the expression of tight junction proteins (ZO‐1 and occludin), ultimately enhancing motor function and reducing neuroinflammation in PD mice.

Cyclooxygenase (COX), also referred to as prostaglandin (PG) H synthase, is responsible for the conversion of arachidonic acid (AA) into PG (Bartels and Leenders 2010). PG plays a crucial role in various biochemical processes that contribute to pain, hyperthermia, inflammation, cytoprotection, and cytotoxicity (Bartels and Leenders 2010). Studies have shown that neuroinflammatory mechanisms involving upregulated COX expression and elevated prostaglandin E2 (PGE2) levels are linked to PD. Clinical trials have indicated that the use of nonsteroidal anti‐inflammatory drugs (NSAIDs) can reduce the risk of developing PD (Bartels and Leenders 2010). Experimental models of PD have further demonstrated an increased susceptibility of COX2 overexpressing neurons to excitotoxicity as well as neuroprotective effects of COX2 inhibition (Minghetti 2004). Acupuncture at GB34 and LR3 points neutralized MPTP toxin‐induced microglial activation in mice and reduced the expression of COX2 and iNOS in the SNpc, as well as protecting dopaminergic neurons from MPTP‐induced neurodegeneration while also reducing dopamine levels in the striatum (Kang et al. 2007). Furthermore, COX2 regulation is independent of microglial activity, so it may be a potential new target for treating PD (Bartels and Leenders 2010).

### 2.6 Regulation of Intestinal Flora

Clinical and animal studies have confirmed that gastrointestinal dysfunction and intestinal pathology precede motor dysfunction in PD (Gershanik 2018). α‐syn can migrate from intestinal neurons to the vagus nerve and subsequently reach the CNS (Gershanik 2018). Thus, the gut plays a crucial role in PD development. The gut‐brain axis refers to the transmission of microbes and their metabolites from the gut to the brain via channels such as the vagus nerve, which conveys neurotransmitters like serotonin and dopamine and regulates hormone levels (Ghaisas et al. 2016; Mulak 2015). The dysregulation of the gut microbiota, by disrupting the gut‐brain axis and elevating both inflammation and gut permeability, represents a potential contributor to the pathogenesis of neurodegenerative disorders. Increasing research suggests that multiple aspects of the gut‐brain axis may play a role in PD (Keshavarzian et al. 2015; Lai et al. 2018). Studies have shown that germ‐free mice receiving fecal transplants from PD patients develop gut microbiota profiles resembling those of their donors and exhibit motor dysfunction when transplanted with fecal matter overexpressing α‐syn from PD patients (Scheperjans et al. 2015). Furthermore, the bacterial endotoxin lipopolysaccharide (LPS) has been found to enhance BBB permeability and stimulate the release of pro‐inflammatory cytokines, including TNF‐α, which contributes to the degeneration of dopaminergic neurons in the SN (Villarán et al. 2010). The inflammatory environment enhances α‐syn aggregation, further activates microglia, and promotes feed‐forward cascades, leading to additional α‐syn aggregation, propagation, and disease progression (Sampson et al. 2016). An 8‐week course of scalp‐abdominal EA was shown to significantly improve both motor and non‐motor symptoms—including sleep, fatigue, and bowel function—as well as overall quality of life in patients with PD (Nazarova et al. 2022). Additionally, the study found that after 8 weeks of acupuncture treatment, species richness, diversity, and taxonomic data decreased significantly. Post‐intervention, the relative abundances of the genera Bacteroides and Parasutterella exhibited significant increases, whereas the abundances of the genera Dialister, Hungatella, Barnesiella, Megasphaera, Allisonella, Intestinimonas, and Moryella were significantly diminished (Nazarova et al. 2022). Similarly, an animal study finds acupuncture reduces abundance of species (Han et al. 2021). This study revealed significant alterations in the relative abundance of Erysipelotrichaceae, Lactobacillus, Bacteroides, Lachnospiraceae, and Ruminococcaceae following EA treatment. Notably, EA treatment markedly mitigated the MPTP‐induced elevation in the relative abundance of Erysipelotrichaceae. Furthermore, Rotarod performance was strongly correlated with Erysipelotrichaceae levels. Previous research has established an association between Erysipelotrichaceae and inflammation, with its relative abundance showing a positive correlation with CD14, TNF‐α, IL‐36, and IL‐6 levels (Kaakoush 2015). Furthermore, prior research has demonstrated that inhibiting cannabinoid receptor 1 can attenuate macrophage inflammatory mediators, including IL‐17 and monocyte chemoattractant protein‐1, by lowering their serum concentrations, along with eotaxin and macrophage inflammatory protein‐1α (Q.‐S. Zhang et al. 2017). This intervention has been shown to ameliorate obesity and metabolic disorders. These improvements were correlated with a reduction in Erysipelotrichaceae within the gut microbiota (Q.‐S. Zhang et al. 2017). Additionally, another study involving HIV‐infected individuals reported a decrease in gut inflammation and a reduction in Erysipelotrichaceae following supplementation with Lactobacillus rhamnosus (Arnbjerg et al. 2018). Therefore, EA at ST36 and GV20 may attenuate inflammation by reducing the high abundance of Erysipelotrichaceae, thereby mitigating the loss of dopaminergic neurons in the SN and alleviating the behavioral deficits induced by MPTP treatment in mice. Another animal experiment, however, demonstrated that acupuncture at GB34 significantly enhanced both the abundance and homogeneity of gut flora in MPTP‐induced PD mouse models (Jang et al. 2020). Notably, the increased abundance of the genus such as Butyricimonas, Holdemania, Aestuariispira, Frisingicoccus, Gracilibacter, and Phocea exhibited a strong correlation with behavioral indices related to anxiety comorbidity, as measured by the open field test, and locomotor deficits (rearing and spinning tests) (Jang et al. 2020). Acupuncture led to an increased abundance of the genus Butyricimonas, a butyrate‐producing bacterium known for its anti‐inflammatory and antioxidant properties (H. Zhang et al. 2022). Moreover, the relative abundance of Holdemania in MPTP‐treated mice decreased following acupuncture treatment. Prior research has indicated that the relative abundance of Holdemania is correlated with motor deficits and anxiety‐related behaviors (Qian et al. 2018; Chung et al. 2019). Additionally, the acupuncture group demonstrated significantly reduced levels of the genus Bacteroides in comparison to both the control and MPTP group (Nazarova et al. 2022). Bacteroides, the largest Gram‐negative genus of bacilli, encompasses species such as Bacteroides fragilis, which are capable of secreting a complex array of pro‐inflammatory neurotoxins, including surface LPS and toxic protein‐hydrolyzing peptides (V. M. Choi et al. 2016). Furthermore, species of Bacteroidales have been shown to stimulate the secretion of TNF‐α from macrophages and monocytes via an LPS‐mediated pathway (Delahooke et al. 1995). Therefore, the reduction of Bacteroidales following acupuncture treatment may partially contribute to the beneficial effects observed in MPTP‐induced PD mice. Subsequent fecal microbiota transplantation experiments in different groups of mice also confirmed that acupuncture‐induced changes in the microbiota may be one of the potential mechanisms underlying its anti‐PD effects.

Dysregulation of lipid‐metabolizing enzymes can directly contribute to PD pathology by altering lipid species (Xicoy et al. 2019). Notably, the signature pathological protein of PD, α‐syn, is known to interact with lipid membranes, and both the endolysosomal system and synaptic signaling pathways in PD are heavily reliant on lipid dynamics (Galper et al. 2022). Furthermore, genetic studies in PD suggest that dysregulated lipid homeostasis may play a role in disease progression (Fanning et al. 2020, Fais et al. 2021). Nutrients, including sugars and fatty acids, play a key role in regulating lipid metabolism (Hu et al. 2024). Recent research indicates that interventions targeting the gut microbiota can enhance metabolic function (Koutnikova et al. 2019). Several reports suggest an association between lipid levels and the composition of gut microbiota, as well as the microbial metabolites they produce (Fu et al. 2015; Aron‐Wisnewsky et al. 2021; Just et al. 2018). The influence of gut flora on host lipid metabolism may be mediated through microbial metabolites, including short‐chain fatty acids, secondary bile acids, trimethylamine, and LPS (Schoeler and Caesar 2019). Additionally, microbiota can alter the lipid composition of host cell membranes, resulting in downstream effects on immunity and metabolism, both local and systemic (Brown et al. 2023). Recent studies have demonstrated that EA at ST25 increases the abundance of beneficial bacteria, such as Lactobacillus, Dubococcus, and Bifidobacterium, in MPTP mice (Hu et al. 2024). This intervention also decreases the abundance of Shigella and Morganella spp. from the genus Pseudomonas and impacts lipid metabolism, including the biosynthesis of unsaturated fatty acids, fatty acids, and bile acids. Notably, dopamine 3‐O‐sulfate, L‐adenosyl‐L‐homocysteine, and S‐adenosyl‐L‐homocysteine, which have been previously reported to be involved in the treatment response of PD patients, are also affected by this treatment (Müller and Kuhn 2006; Harrison et al. 2008). Furthermore, this study identified lipids derived from microbiota, such as β‐glycerophosphoric acid, 4‐hydroxybenzoic acid, and 4‐methoxycinnamic acid. Recent research indicates that these compounds have the potential to effectively modulate the development of PD‐type neuropathy and accumulation of α‐syn (Ho et al. 2019; Ono et al. 2020). Spearman's correlation analysis revealed that the majority of lipids displayed a negative correlation with Escherichia‐Shigella and a positive correlation with Lactobacillus, Dubococcus, and Bifidobacterium. Furthermore, clinical trials have substantiated that Lactobacillus and Bifidobacterium can mitigate inflammation and oxidative stress, as well as ameliorate clinical symptoms in patients with PD (Tamtaji, Naderi Taheri, et al. 2019). In addition, both plasmenylethanolamine and lysoglycerophospholipid levels were elevated following the EA intervention, indicating that this intervention enhanced acetogenin synthesis to mitigate oxidative stress, thereby controlling lipid peroxidation. Correlation analyses further confirmed a strong association between Escherichia‐Shigella and Morganella with motor symptoms and lipid peroxidation in the SN. Therefore, the potential correlation between altered lipid metabolism and gut flora composition may offer novel approaches for targeting the pathological processes of PD. This could represent a significant avenue for acupuncture to influence microbial–metabolomic interactions.

Investigations into the gut microbiota of PD patients reveal a complex and inconsistent landscape (Malkki 2017; Nuzum et al. 2020). This complexity may be attributed to two primary factors. First, microbial ecosystems are significantly influenced by individual dietary habits, and environmental factors may introduce additional regional variability. Second, the inconsistent observations could be partly due to the progressive nature of PD. Additionally, there are substantial differences between the gut microbiota of humans and animals. Consequently, further research is warranted to elucidate these complexities.

## 3 Acupoints in Fundamental Research

The selection of acupoints for the PD animal model was guided by TCM theory, clinical experience, and a review of the relevant medical literature. The acupoints frequently utilized include GB34, ST36, ST25, ST37, LR3, CV4, CV12, GV14, GV16, GV20, GV29, LI4, LI11, HT7, SP6, SP10, SI3, PC7, KI3, SI3, BL62, and NL60 (refer to Table 1 for detailed information). Research employing these acupoints has demonstrated promising outcomes, indicating their potential efficacy in the treatment of PD. Nevertheless, several considerations must be addressed in future studies. First, the predominant combinations of acupoints identified in the literature were GB34 + LR3 and GV20 + GV16, with certain studies incorporating three or more points. However, there is a paucity of research comparing the efficacy of different acupoint combinations. Comparative studies are essential to ascertain the most effective acupuncture treatments. Second, there is a notable scarcity of studies focusing on the comparison of acupuncture frequency and current intensity to provide effective clinical guidelines. Therefore, the duration of acupuncture treatment should be extended to adequately assess its long‐term efficacy. Additionally, in animal studies, the majority of research has concentrated on the motor symptoms of PD, with relatively few investigations exploring the underlying mechanisms of non‐motor symptoms.

**TABLE 1:** Acupuncture for PD in basic researches.

PD model	Intervention	Acupoints	Acupuncture parameters	Frequency and number of treatment sessions	References
MPTP mice	EA	GB34 LR3	50 Hz, 1 mA, 20 min	Once a day for 5 days	(Hsu et al. 2020)
6‐OHDA rats	MA	GU20 GV29 LI4 LR3 DU4	20 min each time	Once a day for 28 days	(Ning et al. 2023)
6‐OHDA mice	EA	KI3	2 Hz, 1 mA, 20 min	Six times, one time every other day	(Tsai et al. 2021)
6‐OHDA rats	MA	ST36	Retained for 20 min	Seven times, one time every other day	(M. S. Lee et al. 2008)
6‐OHDA rats	MA	GB34 LR3 LI4 LI11	Turned at a rate of two spins per second for 15 s, retained for 60 s	Once a day for 13 days	(Kim and Jeon 2014)
6‐OHDA rats	MA	GB34 LR3 ST36 SP10	Retained for 20 min	Seven times, one time every other day	(Y.‐P. Yu et al. 2010)
6‐OHDA rats	MA	CV12 ST25 CV4	Retained for 20 min	Once a day for 15 days	(P. Yu et al. 2025)
MPTP mice	EA	GV16 LR3 ST36	2 Hz, 1 mA, 15min	Once a day for 7 days	(Guo et al. 2024)
MPTP mice	EA	GB34 BL60	2 Hz, 1.5 mA, 100 ms pulse width, 20 min	Once a day, 6 days a week for 3 weeks	(Y. Lee et al. 2023)
MPTP mice	MA	GB34	Rotated for 15 s every 5min, and retained for 10min	Once a day for 12 days	(Zuo et al. 2022)
MPTP mice	MA/EA	PC7	Retained for 15 min/2 and 15 Hz, alternatively, 1 mA, 15 min	Three times, day 3 and day 6 before the first MPTP injection and day 1 after the last MPTP injection	(Duan et al. 2026)
MPTP mice	MA	GB34, LR3	Rotated at a rate of two spins per second for 15 s	14 times, one time every other day	(Fukuda and Egawa 2015)
MPTP rats	EA	GB34, LR3	0/50 Hz, 1 mA, 20 min	Once a day for 5 days	(Lin et al. 2017)
MPTP mice	MA	GB34	Rotated at 2 Hz for 15 s	Once a day for 12 days	(Lei et al. 2016)
Rotenone rats	EA	ST25	2/15 Hz, 2 mA, 20min	Once a day, 5 days a week for 4 weeks	(Hu et al. 2024)
Rotenone mice	EA	DU24 ST25 ST37 LI11	2 Hz, 1 mA, 10 min	Once a day for 8 weeks	(X. Ma, Wang, et al. 2021)
Rotenone rats	EA	ST36	25 Hz, 10 s of “on” time and 90 s of “off” time, 30 min	Once a day for 4 weeks	(Pereira et al. 2021)
MPTP mice	EA	ST36 GV20	2/100 Hz, 0.5–1.0–1.5 mA, 30 min	Once a day for 5 days	(Han et al. 2021)
MPTP mice	EA	chorea trembling control area of the head	2 Hz, 0.2 mA, 15 min	Once a day for 2 weeks	(Geng et al. 2024)
MPTP mice	MA	GB34, LR3	Rotated at a rate of two spins per second for 15 s	14 times, one time every other day	(Fukuda and Egawa 2015)
Rotenone rats	EA	ST25	2/15 Hz, 2 mA, 20 min	Once a day, 5 days a week for 4 weeks	(Sun et al. 2022)
Rotenone rats	EA	GV16 LR3	2 Hz, 20 min	Once a day for 3 weeks	(Kluger et al. 2016)
MPTP mice MPTP+P53 knockout mice	MA	GB34	Rotated at a rate of two spins, per second for 15 s	Once a day for 12 days	(Kong et al. 2018)
MPTP mice	MA	GB34	Rotated at a rate of two spins per second for 15 s	Once a day for 3 days	(Fan et al. 2022)
MPTP mice	MA	GB34 LR3	Rotated at a rate of two spins per second for 15 s	1, 2, 3 treatments once a day	(Gu et al. 2025)
MPTP mice	MA	GB34 LR3	Rotated at a rate of two spins per second for 15 s	14 times, one time every other day	(Liu et al. 2025)
MPTP mice	MA	GB34 LR3	Rotated at a rate of two spins per second for 15 s	14 times, one time every other day	(Gu et al. 2025)
MPTP mice	EA	GB34	2/100 Hz, 1 mA, 20 min	Once a day for 12 days	(Gao et al. 2024)
MPTP mice	EA	ST36 SP6	0/100 Hz, 1–1.25–1.5 mA, 30 min	Once a day, 5 days a week for 2 weeks	(H. Wang et al. 2013)
MPTP mice	MA	GB34	Rotated at a rate of two spins per second for 15 s, retained for 10 min	Once a day for 12 days	(Tian et al. 2016)
MPTP mice	MA	GB34 ST36	Rotated at a rate of two spins per second for 30 s	Once a day for 12 days	(Jang et al. 2020)
MPTP mice	MA	GB34	Rotated at a rate of two spins per second for 15 s	Once a day for 12 days	(Fukuda et al. 2016)
MPTP mice	MA +Chunggan formula	GB34	Rotated at a rate of two spins per second for 30s	Once a day for 8 days	(Shaosong et al. 2016)
MPTP mice	MA	GB34 HT7	Rotated at a rate of two spins per second for 30 s	Once a day for 12 days	(Yu et al. 2019)
MPTP mice	EA	ST36 SP6	0/100 Hz, 1–1.25–1.5 mA, 30 min	Once a day, 5 days a week for 2 weeks	(H. Wang et al. 2011)
MPTP mice	MA	GB34 SI3	Rotated at a rate of two spins per second for 30 s	Once a day for 12 days	
MPTP mice	MA	GB34 LR3	Rotated at a rate of two spins per second for 15 s	Three times, treatment at 1,3,7 days after MPTP injection	(Kang et al. 2007)
6‐OHDA mice	MA	GB34	Rotated at a rate of two spins per second for 15 s	Once a day for 10 days	
MPTP mice	MA	GB34	Rotated at a rate of two spins per second for 15 s	Once a day for 12 days	
MFB‐axo rats	EA	GV14 GV20	2/100 Hz, 1–2–3 mA, 30 min	Once a day for 24 days	(Liang et al. 2002)
MFB‐axo rats	EA	GV14 GV20	2/100 Hz, 1–2–3 mA, 30 min	Once a day, 6 days a week for 2 or 4 weeks	(Liang et al. 2003)
MFB‐axo rats	EA	GV20 GV29	2 Hz, 1.5 mA, 10 min	Once a day for 7 days	

Abbreviations: 6‐OHDA, 6‐hydroxydopamine; EA, electroacupuncture; MA, manual acupuncture; MFB‐axo, medial forebrain bundle axotomy; MPTP, 1‐methyl‐4‐phenyl‐1,2,3,6‐tetrahydropyridine.

## 4 Clinical Studies: Acupoint Selection, Controversies Regarding Efficacy, and Future Directions

The selection of acupoints in clinical studies is primarily based on TCM theory, clinical experience, and existing literature. Table 2 summarizes the acupoints and their combinations used for different symptoms of PD, such as gait disorders, fatigue, constipation, anxiety, sleep disturbances, dysphagia, and pain. Commonly used acupoints include GV20, ST36, GB34, LR3, SP6, LI4, PC6, HT7, and KI3. In terms of stimulation methods, both MA and EA have been used in clinical studies. For EA, the frequency is mostly concentrated between 2 and 100 Hz. The single session needle retention time is usually 20–30 min, treatment frequency ranges from once a week to once a day, and the course of treatment is mostly 4–12 weeks.

**TABLE 2:** Acupuncture for PD in clinic researches.

PD symptom	Intervention	Acupoints	Acupuncture parameters	Frequency and number of treatment sessions	References
Gait	MA	Tender points	10 min	Once a week for 4 weeks	(Fukuda and Egawa 2015)
Gait	MA/EA	Foot Motor Sensory Area, Balance Area, GV20, GV14, LI4, ST36, GB34, BL40, SP6, KI3, LR3	4 or 100 Hz, 30min	Once a week for 3 weeks	(Lei et al. 2016)
Gait	MA	ST34, BL57, HT3, HT7, KI3, KI7, SP4	30 min	Single session only	(Pereira et al. 2021)
Gait	MA	Motor area, Chorea‐tremor, Controlled area, Balance area	20 min (with simultaneous walking)	Single session only	(Sun et al. 2022)
Fatigue	MA	GV 20, GV 24, LI 10, HT 7, ST36, SP 6, LI10	30 min, twisted three times to the right	Twice a week for 6 weeks	(Kluger et al. 2016)
Fatigue	MA	PC6, LI4, ST36, SP 6, KI 3, CV 6	20 min	Twice a week for 5 weeks	(Kong et al. 2018)
Constipation	EA	GV2, GB5, EX‐HN1, GB6, LI11, LI4 GB34, ST36, SP6, KI3, LR3, ST25, SP14, ST37	10/50 Hz, 1–10 mA, 30min	Three times a week for 12 weeks	(K. Li et al. 2023)
Anxiety	MA	GV24, GV29, GV21, GV19, HT7, SP6	Needle twisting for 1 min at 180–200 rpm, needle retention for 30 min	Once a day, three times a week for 8 weeks	(Fan et al. 2022)
Sleep disturbance	EA	PC6, HT7, EX‐HN22, GV29, GV20, LI4, SP6, KI3, LR3, GB34, GV21, GB5, EX‐HN1, GB6, GV20, GV29	30 Hz, 1–5.0 mA, 30 min	Three times a week, one time every other day for 8 weeks	(Gu et al. 2025)
Sleep disturbance	MA	BL62, KI6, PC7, HT7, EX‐B2	30 min	Three times a week for 4 weeks	(Liu et al. 2025)
Dysphagia	EA	Ex‐HN‐10 Ex‐HN‐11	Needle twisting 12 times (1–2 min), needle retention for 20 min, repeat twisting before removal; 30–80 Hz, up to 25 mA	Once a day for 30 days	(Gao et al. 2024)
Dysphagia	MA	ST36, SP6, LR3, LI4, LI11, GB20, BL18, BL23	10–15 min	Single session only	(Fukuda et al. 2016)
Musculoskeletal pain	EA	GV20, CV6, LU7, SI19, LI15, LI11, SP10, ST36	2 Hz/100 Hz, 30min	Once a day, five times a week for 4 weeks	(Shaosong et al. 2016)
Pain	MA	GV20, 77.18, GB34	30 min	1–3 times a week, at least 1 day apart, for 8 weeks	(Yu et al. 2019)

It is worth noting that the clinical efficacy of acupuncture for PD remains somewhat controversial. Some studies, especially systematic reviews published before 2020, indicated that in randomized controlled trials (RCTs) comparing acupuncture with sham acupuncture, there were no significant differences between the two groups in outcomes such as Unified PD Rating Scale (UPDRS) scores, quality of life, and depression. The systematic review by M. S. Lee et al. (2008) included three RCTs comparing acupuncture with sham acupuncture and found no superiority of acupuncture. Similarly, Kim and Jeon concluded that the evidence for the efficacy of acupuncture in PD was not convincing (Kim and Jeon 2014). However, the research trend in recent years presents a different picture. Most clinical studies and systematic reviews published after 2020 have reported significant improvements in both motor and non‑motor symptoms of PD with acupuncture. A 2025 meta‑analysis including 50 RCTs (n = 3248) showed that acupuncture combined with conventional medication was significantly superior to conventional medication alone in all UPDRS dimensions, with the most pronounced effect on treatment‑related motor complications (P. Yu et al. 2025).

The differences in efficacy observed over this time period may be due to several factors. First, early studies may not have fully mastered the standardization of acupuncture procedures and acupoint selection, whereas recent studies have paid more attention to the integration of standardization and individualization in treatment protocols. Second, most early negative studies were conducted by non‑Chinese researchers, whose understanding of TCM theory and acupuncture manipulation may have been limited, potentially preventing the intervention from fully realizing the potential effects of acupuncture. Furthermore, the methodological rigor of clinical studies on acupuncture for PD has improved in recent years, with some studies adopting stricter RCT designs and more standardized efficacy evaluation systems, thereby providing higher‑quality evidence to confirm the efficacy of acupuncture. Nevertheless, it must be acknowledged that the overall methodological quality of existing studies remains insufficient. A 2020 systematic review evaluated 11 systematic reviews/meta‑analyses using the AMSTAR‑2 tool and found all to be of critically low quality (J. Huang et al. 2020). According to the GRADE evidence quality grading, among the evaluated outcomes, 20 were of very low quality, 9 of low quality, and 3 of moderate quality, with no high‑quality evidence (J. Huang et al. 2020). Therefore, the clinical efficacy of acupuncture still requires further verification through well‑designed, adequately powered multicenter RCTs.

Although existing clinical studies demonstrate the potential value of acupuncture in improving various symptoms of PD, several issues remain to be addressed. First, the combination of acupoints in clinical studies is highly diverse. Although some studies have developed relatively fixed protocols for specific symptoms (e.g., gait disorders often treated with the motor area and balance area of the scalp combined with distal acupoints on the limbs), there is a lack of comparative studies evaluating the efficacy of different acupoint combinations. Consequently, it remains unclear which acupoint combinations are optimal for different symptoms. Second, acupuncture parameters (such as frequency, intensity, needle retention time, treatment frequency, and total course) vary considerably across studies, and there is a lack of systematic research on standardization and dose‑response relationships, which limits the reproducibility and generalizability of clinical procedures. Recent studies suggest that using more than 10 acupoints per session, moderate single‑session needle retention time, and three treatments per week may be the optimal parameter combination (P. Yu et al. 2025). In addition, most existing studies focus on short‑term improvements in single symptoms (4–12 weeks), and long‑term efficacy and safety monitoring data for acupuncture are relatively scarce (Duan et al. 2026). In particular, safety analyses for patients with comorbidities remain insufficient. Therefore, future research should focus on optimizing acupoint combinations, conducting dose‑effect analyses of acupuncture parameters, and extending follow‑up periods to evaluate long‑term efficacy and safety—specifically, follow‑up of at least 12 months is recommended, with standardized documentation of adverse events such as skin irritation and infection, as well as subgroup analyses for patients with comorbidities, in order to objectively assess the safety profile of acupuncture. This would provide a more solid evidence base for the clinical translation of acupuncture therapy for PD.

## 5 Conclusion

**FIGURE 1:**
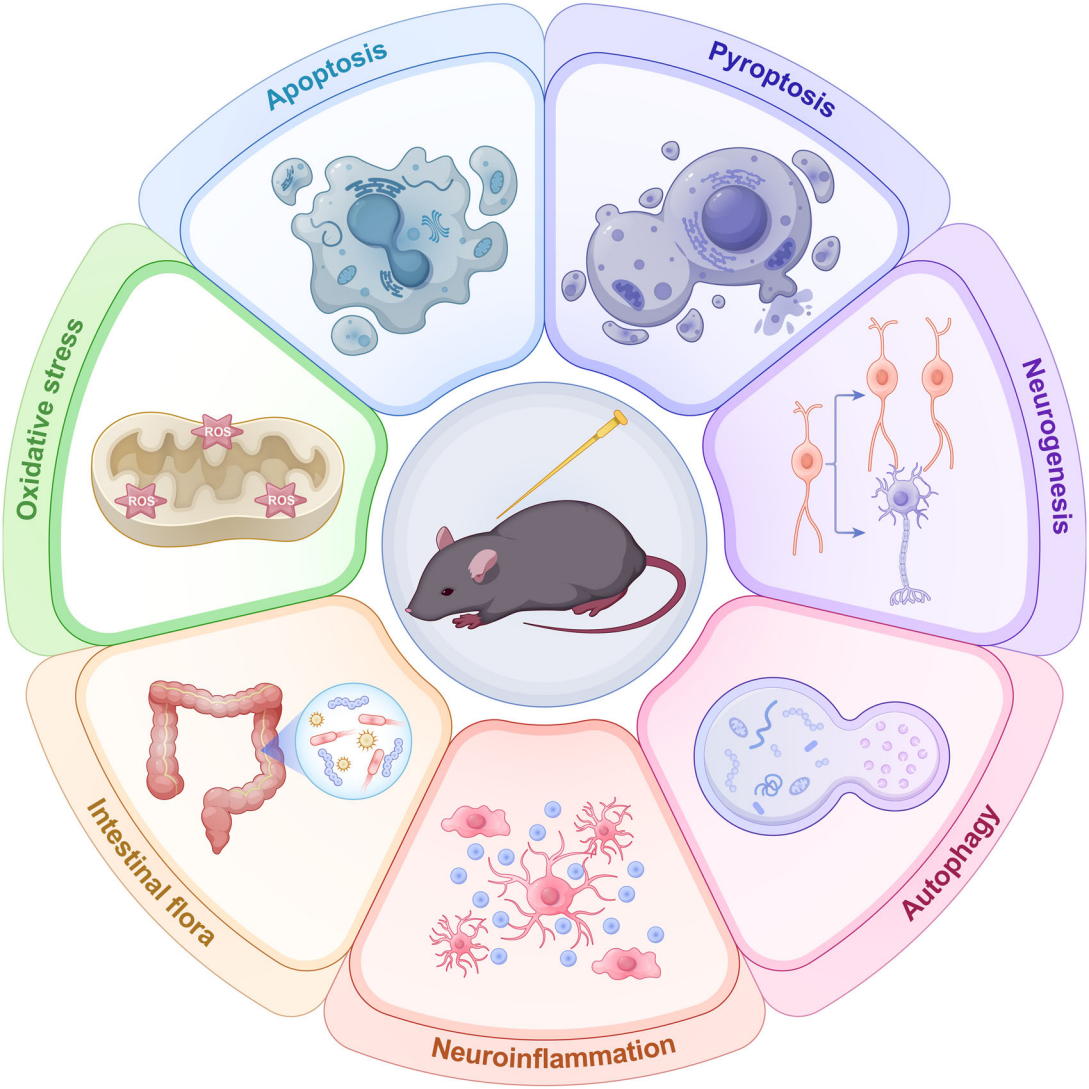
The mechanism of acupuncture treatment of PD involves oxidative stress, intestinal flora, neuroinflammation, autophagy, neurogenesis, pyroptosis, and apoptosis.

A substantial body of clinical, preclinical, and fundamental research has established the efficacy of acupuncture in treating PD. The primary benefits include symptomatic relief and the reversal of dopaminergic neurodegeneration during the early stages of the disease. Integrating acupuncture with anti‐Parkinsonian pharmacotherapy may potentiate the therapeutic effects of the medications, thereby allowing for a reduction in dosage and minimizing associated adverse effects. Contemporary evidence corroborates the effectiveness of both EA and traditional acupuncture in managing PD in both human patients and animal models. These therapies influence various pathways, including apoptosis, autophagy, oxidative stress, gut microbiota, neuroinflammation, and neurogenesis (summarized in Figure 1). A comprehensive understanding of the neuroprotective effects of EA in PD will provide a theoretical foundation for its therapeutic application and facilitate the broader implementation of EA therapy.

However, current research exhibits several limitations: (1) Given that PD is a chronic condition, it is imperative to investigate the long‐term effects of EA on the animal model to accurately predict its therapeutic efficacy. (2) Acupoints may differ between animals and humans, potentially resulting in varied effects and molecular changes. Consequently, caution is warranted when extrapolating these findings to humans. (3) The majority of studies on acupuncture or EA utilizing animal models of PD have predominantly focused on preventive effects rather than enduring reversal effects. To substantiate the long‐term efficacy of acupuncture in PD, it is imperative to conduct studies following the confirmation of dopaminergic neuronal degeneration and the onset of motor dysfunction. (4) At present, research utilizing animal models predominantly concentrates on the motor symptoms associated with PD, while investigations into non‐motor symptoms remain comparatively scarce. (5) The mechanism of action of EA in the context of PD is both extensive and heterogeneous, with the interactions among various molecular pathways remaining inadequately elucidated. Furthermore, there is a lack of standardization in the acupoints utilized across different studies, and the comparative efficacy of various combinations of these points has not been systematically evaluated. Consequently, additional research is imperative to elucidate the therapeutic potential of acupuncture in PD and to uncover its underlying mechanistic pathways.

## Author Contributions

**Xi‐Chen Wu**: conceptualization and writing – original draft. **Ping Yin**: conceptualization, supervision and conceptualization. **Yu‐Chen Ying**: conceptualization, investigation and methodology. **Yi‐Yue Dong**: conceptualization, investigation and methodology. **Yue‐Lai Chen**: conceptualization, funding acquisstion.

## Funding

This work was supported by the Clinical Incubation Program of the National Medical Center of LongHua Hospital to Shanghai University of Traditional Chinese Medicine (GY202201), the Shanghai Shenkang Hospital Development Center demonstration research ward construction project (SHDC2022CRW006), the Shanghai Pudong New District Health Industry Special Project (PW2021E‐01), the Shanghai Leading Talent Project (202013) and the Shanghai Municipal Health Commission Health Industry Clinical Research Special Top Project (202340110) and the Construction of Traditional Chinese Medicine Inheritance and Innovation Development Demonstration Pilot Projects in Pudong New Area—High‐Level Research‐Oriented Traditional Chinese Medicine Hospital Construction (YC‐2023‐0901).

## Conflicts of Interest

The authors declare no conflicts of interest.

## Data Availability

No data were used for the research described in the article.
